# Whole genome sequencing and phylogenetic analysis of six SARS-CoV-2 strains isolated during COVID-19 pandemic in Tunisia, North Africa

**DOI:** 10.1186/s12864-021-07870-1

**Published:** 2021-07-14

**Authors:** Wasfi Fares, Anissa Chouikha, Kais Ghedira, Meriam Gdoura, Dorra Rezig, Sondes Haddad Boubaker, Imen Ben Dhifallah, Henda Touzi, Walid Hammami, Zina Meddeb, Amel Sadraoui, Nahed Hogga, Imen Abouda, Aurélia Kwasiborski, Véronique Hourdel, Guillain Mikaty, Valérie Caro, Jean-Claude Manuguerra, Nissaf Ben Alaya, Henda Triki

**Affiliations:** 1 Laboratory of Clinical Virology, Institut Pasteur, University Tunis-El Manar, Tunis, Tunisia; 2 Laboratory of Bioinformatics, Biomathematics and Biostatistics (BIMS), Institut Pasteur Tunis, Tunis, Tunisia; 3 Laboratory for Urgent Response to Biological Threats (CIBU), Environment and Infectious Risks (ERI) research and expertise unit, Institut Pasteur, Paris, France; 4 National Observatory for New and Emerging Diseases, Ministry of Health, Tunis, Tunisia; 5 Faculty of Medicine, University Tunis-El Manar, Tunis, Tunisia

**Keywords:** Whole genome sequencing, SARS-CoV-2, SNPs, Genotype, Tunisia

## Abstract

**Background:**

In Tunisia a first SARS-CoV-2 confirmed case was reported in March 03, 2020. Since then, an increase of cases number was observed from either imported or local cases. The aim of this preliminary study was to better understand the molecular epidemiology and genetic variability of SARS-CoV-2 viruses circulating in Tunisia and worldwide.

**Methods:**

Whole genome sequencing was performed using NGS approach on six SARS.

CoV-2 highly positive samples detected during the early phase of the outbreak.

**Results:**

Full genomes sequences of six Tunisian SARS-CoV-2 strains were obtained from imported and locally transmission cases during the COVID-19 outbreak. Reported sequences were non-identical with 0.1% nucleotide divergence rate and clustered into 6 different clades with worldwide sequences. SNPs results favor the distribution of the reported Tunisian sequences into 3 major genotypes. These SNP mutations are critical for diagnosis and vaccine development.

**Conclusions:**

These results indicate multiple introductions of the virus in Tunisia and add new genomic data on SARS-CoV-2 at the international level.

## Background

Human Coronaviruses (HCoV’s) are members of the subfamily *Coronavirinae* in the family of *Coronaviridae* in the order of *Nidovirales*. Since 1960s, six different HCoV’s were identified; two belongs to the genus *Alphacoronavirus* (229E, NL63) and four to the genus *Betacoronavirus* (HKU1 and OC43, SARS-CoV, MERS-CoV). Similarly to other *coronaviruses*, SARS-CoV-2 is an enveloped, single-stranded positive-sense RNA (~ 29,900 nt) with 5′-cap structure and 3′-poly-A tail [1].

In December 2019, a novel Human Coronavirus (HCoV) associated to severe acute respiratory syndrome was discovered in the city of Wuhan, Hubei province, China and was later named SARS-CoV-2 [2, 3]. The outbreak of the coronavirus disease (COVID-19) spread further worldwide and the World Health Organization (WHO) officially declared the pandemic aspect of the COVID-19 epidemic on March 12th, 2020 [4]. The WHO report dated on June 21st, 2020 confirmed 8,708,008 cases of SARS-CoV-2 with 461715deaths from 215 countries and territories [5].

The Tunisian government, through the Ministry of Health (MoH) and the National Observatory of New and Emerging Diseases, reviewed and initiated multisectoral measures to deal with the COVID-19 epidemic. The national strategy aims early detection of imported cases, isolation of confirmed cases as well as suspected cases. Moreover, the MoH commit to develop diagnostic and treatment procedures in order to limit the spread of the SARS-CoV-2 within the population and avoid exceeding of the health system capacity. These measures allowed the Tunisian authorities to identify the first SARS-CoV-2 in March 03, 2020 [6]. As of June 7th, a total of 1087 COVID-19 cases, including 286 imported and 801 locally transmitted cases, have been diagnosed and tested positive for SARS-CoV-2 with 49 deaths [7].

This work reports the first whole genome sequences of the SARS-CoV-2 viruses isolated in Tunisia during the early phase of the outbreak. Phylogenetic comparison with whole genome sequences reported from other countries and single-nucleotide polymorphism (SNPs) were assessed.

## Results

**Fig. 1:**
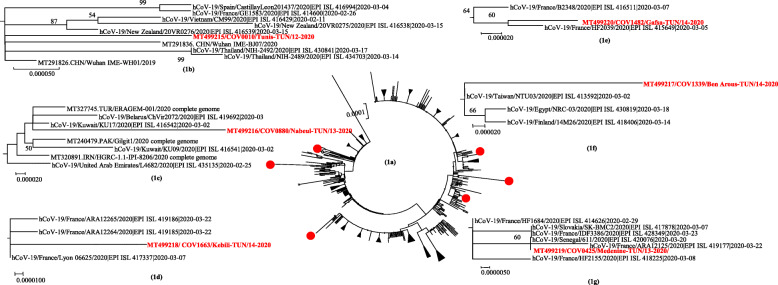
Phylogenetic tree comparing the six Tunisian SARS-CoV-2 with 268 previously published sequences. The tree was performed using the neighbor joining method and the kimura-2 parameter model. Topology was supported by 1000 bootstrap replicates. For better clarity of the tree, lineages from the same country were collapsed and bootstrap values lower than 50 were not indicated (**a**). Phylogenetic clusters including SARS-CoV-2 Tunisian genome sequences were detailed in (**b** to **g**). The sequences reported in this study are shown in red bold, and indicated by the accession number, laboratory code, district, country code, week and year of isolation. The sequences downloaded from GISAID and NCBI database are indicated by their accession number followed by the country code and the year of isolation

The six Tunisian sequences obtained were 29,541 to 29,825 nucleotide-long and covered the whole coding region and more than 99.1% of the genome. The totality of complete genome sequences available at writing time, of SARS-CoV-2 were downloaded from the GISAID (http://www.gisaid.org/) and the NCBI (http://www.ncbi.nlm.nih.gov/genbank) databases. All the repeated sequences as well as the sequences with missing regions have been excluded. The 268 selected sequences originated from 61 different countries worldwide. The phylogenetic tree in Fig. 1, shows that Tunisian SARS-CoV-2 sequences were scattered independently of each other in different clusters including strains mainly from Europe and Asia (Fig. 1a). Three out of six Tunisian sequences clustered with sequences from France: one case came from France (COV0425, Fig. 1g) and two were locally infected (COV1482 and COV1663, Fig. 1d and e). The sequence COV1339 was isolated from a case coming from Turkey, it grouped with sequences originated from Taiwan, Egypt and Finland (Fig. 1f). Sequence COV0010 was from a case with recent travel to Vietnam and clustered with sequences from Thailand, Vietnam, China and Japan (Fig. 1b). The last identified sequence COV0880 was from a locally infected individual and grouped with sequences from Turkey, Belarus and Kuwait (Fig. 1c).

The Tunisian SARS-CoV-2 retrieved full genome sequences alignment with the Wuhan reference sequences (NC045512) were used for the investigation of the SNPs and the resulting variable amino-acids composition throw genome detective web server (https://www.genomedetective.com/); the obtained results are shown in Table 1.

**Table 1:** Single nt polymorphisms (SNPs) deduced by comparison of six Tunisian whole genome SARS-CoV-2 sequences with reference SARS-CoV-2 NC045512

**Gene**		**nsp2**					**nsp 3**						**nsp4**				**nsp6**	**nsp7**	**nsp8**	**nsp12**			
**Nucleotide position**		1059	1397	1666	2113	2416	3037	3896	4288	5557	7420	8371	8782	9479	9514	10,046	11,083	11,935	12,555	13,495	14,408	15,324	16,178
Cov0010	SNPs mutations									**T > C**			*C > T*							*C > T*			
	Proteins mutations									**G946G**			*S75S*							*L19F*			
Cov0880	SNPs mutations		**G > A**						**G > T**					*G > T*	**A > G**	*G > A*	**G > T**		**A > C**				
	Proteins mutations		**V198I**						**E523D**					*G309C*	**L320L**	*V498I*	**L37F**		**E155A**				
Cov1339	SNPs mutations				*C > T*		**C > T**														**C > T**		*C > T*
	Proteins mutations				*L436L*		**F105F**														**P323L**		*S913L*
Cov1663	SNPs mutations						**C > T**	**G > T**			**C > T**							**A > C**			**C > T**	**C > T**	
	Proteins mutations						**F105F**	**V393F**			**I1567I**							**Q31H**			**P323L**	**N628N**	
Cov0425	SNPs mutations					C > T	**C > T**					**G > T**									**C > T**		
	Proteins mutations					Y537Y	**F105F**					**Q1884H**									**P323L**		
Cov1482	SNPs mutations	**C > T**		**T > C**			**C > T**														**C > T**		
	Proteins mutations	**T85I**		**F/287F**			**F105F**														**P323L**		
**Gene**		**nsp13**	**nsp14**			**nsp15**	**S**								**Orf3a**		**Orf8**		**N**				
**Nucleotide position**		17,690	18,523	18,877	19,525	20,032	21,648	22,210	22,397	22,424	22,435	22,468	22,503	23,403	25,411	25,563	27,896	28,144	28,346	28,347	28,835	28,878	
Cov0010	SNPs mutations											*G > T*						**T > C**				*G > A*	
	Proteins mutations											*T302T*						**L84S**				*S202N*	
Cov0880	SNPs mutations								*T > A*						**A > C**		**G > T**				*T > C*		
	Proteins mutations								*Y279N*						**I7L**		**M1I**				*S188P*		
Cov1339	SNPs mutations	**C > T**		**C > T**	*G > T*			*C > T*		*G > A*	*T > C*		*A > G*	**A > G**		**G > T**							
	Proteins mutations	**S485L**		**L280L**	*D496Y*			*P216L*		*A288T*	*C291C*		*Q314R*	**D614G**		**Q57H**							
Cov1663	SNPs mutations		**G > T**			**C > T**	**C > T**							**A > G**									
	Proteins mutations		**V162L**			**R138C**	**T29I**							**D614G**									
Cov0425	SNPs mutations													**A > G**		**G > T**							
	Proteins mutations													**D614G**		**Q57H**							
Cov1482	SNPs mutations													**A > G**		**G > T**			**G > T**	**G > T**			
	Proteins mutations													**D614G**		**Q57H**			**G25F**	**G25F**			

(In bold and underlined: confirmed mutations with more than 2500 reads. In italics: validated mutations with less than 2500 reads)

The average and the Min/Max depth of coverage for the six described isolates was as follows, Cov0010: 12545 (0/35320), Cov0880: 9339 (0/41049), Cov1339: 10456 (0/35132), Cov1663: 13504 (0/31108), Cov0425: 12982 (0/32281) and Cov1482: 12360 (0/30130). To properly present the mutants described in Table 1, confirmed mutations with more than 2500 reads are written in bold and underlined, and validated mutations with less than 2500 reads are written in italics.

The six Tunisian SARS-CoV-2 sequences were non identical with 0.1% nucleotide divergence between each other. Compared to the Wuhan (NC045512) reference, the reported Tunisian SARS-CoV-2 sequences showed several synonymous and non-synonymous mutations, distributed over the entire genome. A total of 43 SNP mutations were identified with 27 and 16 SNPs in the non-structural and structural region respectively. Each sequence showed a different SNPs profile; the number of detected SNPs in each sequence ranged between 6 and 13. Most of them were located in the non-structural region affecting 08 out of the 16 non-structural genes (Table 1). In the structural region, most of SNPs were detected in the S gene encoding for the spike protein.

## Discussion

In this work, the full genomes sequences of six SARS-CoV-2 strains were obtained from RT-PCR positive samples collected in Tunisian patients between March and April 2020. Three cases had a recent travel history, one coming from Vietnam, one from Turkey and one from France; while the three remaining cases were infected locally through horizontal transmission. The six sequences were non-identical with 0.1% nucleotide divergence between each other and had several nucleotide changes compared to the first published COVID-19 sequence from Wuhan, China (NC045512). Phylogenetic analysis including representative sequences from 61 countries split the six sequences from Tunisia into 6 different clusters, together with sequences from various other countries. These results indicate multiple introductions of the SARS-CoV-2 virus in Tunisia.

According to recently published results by Yin in 2020, four major genotypes were suggested depending on the positions of SNPs [8]. The SNPs here reported in the positions 3037, 8782, 11,083, 14,408, 23,403 and 28,144 allowed us to classify the Tunisian sequences into 3 distinct genotypes among the four major genotypes described: Genotype I (COV0880), genotype III (COV0010) and Genotype IV (COV0425, COV1339, COV1482 and COV1663). Indeed, these SNPs were identified as the most frequent mutations described since of the beginning of the pandemic in comparison with the Wuhan reference SARS-CoV-2 genome NC045512.2 [8, 9]. The highly frequent SNP mutations was previously described in the S protein, RNA polymerase, RNA primase, and nucleoprotein, which are critical proteins for diagnosis and vaccine development [8, 10].

Further complementary studies including clinical status of COVID-19 cases will be of a great interest to better understand the impacts of these SNP mutations on the transmissibility and pathogenicity of SARS-CoV-2.

## Conclusions

Analysis of SARS-CoV-2 sequences from the maximum of affected countries and in different phases of the COVID-19 outbreak is crucial to understand the virus transmission and its genetic evolution through time. This preliminary study reports the first SARS-CoV-2 whole genome sequences from Tunisia, North Africa. Further sequences from different clinical and epidemiological settings are still needed to monitor the molecular epidemiology of SARS-CoV-2 in the country and worldwide.

## Methods

Detection of SARS-CoV-2 infection was performed on nasopharyngeal swabs samples by specific real time reverse transcription polymerase chain reaction (RT-PCR) according to the WHO approved protocol published by Corman et al. and detecting specific sequences in the E, N and RdRp genes [11]. A total of 6 SARS-CoV-2 highly positive samples were assessed for whole genome sequencing using the NGS approach. The respective COVID-19 cases were detected between March and April 2020. They originated from six different districts of Tunisia. Three cases had recent travel history and three were infected locally.

A total of 140 μl of each nasopharyngeal sample was used for RNA extraction via the Qiamp viral RNA mini kit (Qiagen, Hilden, Germany). Reverse transcription of the whole genome was performed by The ProtoScript II First Strand cDNA synthesis kit (New England Biolabs, USA) using random hexamers. For the multiplex PCR approach, the general method described in (https://artic.network/nCoV-2019) was applied using Version 3 of amplicon set. After 40 amplification cycles, the PCR products were cleaned-up with AMPure XP magnetic beads (Beckman Coulter, USA) and quantified with the Qubit 2.0 fluorometer (ThermoFisher Scientific, USA).

The Nextera DNA Flex Prep kit (Illumina, USA) was used for library preparation and thereafter qualified on an Agilent Technologies 2100 Bioanalyser using a high-sensitivity DNA chip following the manufacturer’s instructions. Generated libraries were sequenced using the MiSeq System (Illumina, USA) providing 2 × 250 bp read length data.

To remove low quality reads, trim off low-quality and contaminant residues and filter out duplicated reads, fqCleaner v.0.5.0 was used, with Phred quality score of 28. Filtered reads were mapped against SARS-CoV-2 reference (NC045512) using Burrows-Wheeler Aligner MEM algorithm (BWA-MEM) (v0.7.7). SAM tools were used to sort BAM files, to generate alignment statistics and to obtain coverage data. Whole genome sequences of the six SARS-CoV-2 Tunisian strains were submitted to NCBI database under accession number MT499215 to MT499220.

Multiple sequences alignment was performed using Multiple Sequence Comparison by Log- Expectation (MUSCLE) software implemented in Molecular Evolutionary Genetics Analysis software (MEGA) version 7 [12]. Phylogenetic analysis was performed using the neighbor-joining analysis method and the Kimura 2-parameter model with 1000 bootstrap replications. Genetic distances were calculated using the p-distance method.

## Data Availability

The six SARS-CoV-2 Tunisian strains sequences, obtained in the current study, were submitted to NCBI database and the accession number with the corresponding strains is as follow MT499215, MT499216, MT499217, MT499218, MT499219 and MT499220 for strains COV0010, COV0880, COV1339, COV1663, COV0425 and COV1482, respectively (https://www.ncbi.nlm.nih.gov/nuccore/MT499215,MT499216,MT499217,MT499218,MT499219,MT499220). The raw reads datasets generated and analyzed during the current study were submitted to the NCBI Sequence Read Archive (SRA) repository and freely available under following accession numbers SRX11188766 to SRX11188771 for the corresponding whole genome sequence MT499215 to MT499220 respectively. The SARS-CoV-2 isolate Wuhan-Hu-1 sequence used us a reference strain is available on the https://www.ncbi.nlm.nih.gov/nuccore/NC_045512.

## Abbreviations

COVID: Corona Virus Disease
GISAID: Global Initiative on Sharing Avian Influenza Data
MEGA: Molecular Evolutionary Genetics Analysis software
MUSCLE: Multiple Sequence Comparison by Log- Expectation
NGS: New generation Sequencing
RNA: Ribo-Nucleic-Acid
RT-PCR: Reverse transcription polymerase chain reaction
SARS-CoV-2: Severe acute respiratory syndrome coronavirus 2
SNPs: Single Nucleotides Polymorphism
SRA: Sequence read Archive
